# Exogenous regulators enhance strawberry yield under heat stress conditions

**DOI:** 10.1038/s41598-026-35730-z

**Published:** 2026-01-16

**Authors:** Najra-Tan-Nayeem Salwa, Sadia Shabnam Swarna, Sharifunnessa Moonmoon

**Affiliations:** Department of Crop Botany and Tea Production Technology, Faculty of Agriculture, Sylhet Agricultural University, Sylhet, 3100 Bangladesh

**Keywords:** Heat stress, Strawberry (*Fragaria × ananassa*), Kaolin spray, BARI strawberry-3, Fruit quality, Physiology, Plant sciences

## Abstract

Climate change poses a growing threat to strawberry cultivation, a crop highly sensitive to elevated temperatures. Developing sustainable strategies to mitigate heat induced stress is crucial for maintaining yield and fruit quality. This study evaluated the effectiveness of different exogenous chemical treatments in enhancing heat stress tolerance across three strawberry genotypes RABI-3, BARI Strawberry-2, and BARI Strawberry-3 under prolonged summer conditions in Bangladesh. A two-factorial completely randomized design (CRD) was conducted from February 2024 to April 2025 with six treatments: control (water), abscisic acid (5 ppm), calcium chloride (10 mM), kaolin (5%), melatonin (10 ppm), and molasses (5%). Vegetative, physiological, biochemical, and yield parameters were analyzed, and treatment effects were statistically tested at 5% significance level. Kaolin application significantly improved vegetative growth (plant height and leaf number increased by 15–25% compared to the control) and enhanced photosynthetic activity and antioxidant capacity. The kaolin treated plants also showed a 20–30% yield increase with better fruit size, total soluble solids, and vitamin C content. Among genotypes, BARI Strawberry-3 demonstrated the highest tolerance and yield stability under high-temperature stress.

These findings suggest that kaolin spray may offer a cost effective and environmentally sustainable approach to mitigating heat stress in strawberries, with BARI Strawberry-3 representing a promising genotype for future commercial cultivation in warming agro-climatic zones.

## Introduction

Strawberry (*Fragaria × ananassa Duch*.) is a commercially important soft fruit crop belonging to the Rosaceae family, widely cultivated across temperate and, increasingly, tropical and subtropical regions due to the development of day neutral cultivars^1,2^. It is prized for its rich flavor, aroma, and high nutritional value, and is a significant source of antioxidants, phenolic compounds, and vitamin C, with levels even higher than lemon^3,4^. Global strawberry production exceeds 10.5 million tons annually, with China alone contributing over 40%^5^. The fruit is consumed fresh or processed into value-added products such as juices, jams, syrups, and frozen desserts, contributing significantly to the global economy^6^.

In tropical climates, such as Bangladesh, strawberry cultivation faces considerable constraints due to seasonal heat stress. The species performs optimally at day temperatures of 22–23 °C and night temperatures of 7–13 °C^7^, which only occur during winter months in this region^8^. Strawberry propagation in these environments relies almost exclusively on vegetative methods using runners or suckers, requiring farmers to maintain mother plants through the summer^9,10^. This exposes plants to high temperatures exceeding 30 °C, leading to physiological stress, reduced vegetative vigor, and in severe cases, complete mortality.

Heat stress above 26 °C inhibits strawberry growth and development^11^, causing visible symptoms such as leaf wilting, scorching, and physiological disruptions such as damage to Photosystem II, thylakoid membrane degradation, increased reactive oxygen species (ROS) accumulation, electrolyte leakage, and impaired membrane integrity^12^. These changes ultimately impair pollen viability, fruit set and quality^13^, making strawberry production increasingly unreliable^14^.

To address these challenges, exogenous application of chemical compounds has emerged as a promising strategy to improve plant resilience under abiotic stress conditions. Abscisic acid (ABA) enhances root architecture and water uptake and promotes antioxidant activity and proline accumulation, aiding osmotic adjustment^15,16^. Melatonin mitigates oxidative stress by scavenging ROS and modulating the expression of antioxidant enzymes^17,18^. Foliar application of kaolin reduces leaf temperature^19^ and transpiration by increasing reflectance and decreasing stomatal density, thereby improving relative water content and chlorophyll retention^20,21^. Calcium chloride is known to stabilize cell membranes and enhance enzymatic antioxidant defenses^22^, while organic amendments like molasses have been shown to boost antioxidant activity and reduce oxidative damage^23^.

While there have been studies into heat tolerance mechanisms for strawberries, few studies focused on vegetative stage resilience under tropical field conditions, where maintaining mother plants through high temperature periods is critical for runner propagation and subsequent yield. Furthermore, there is limited research available that has evaluated the effects of multiple exogenous regulators, such as plant growth regulators, across genotypes, which represents an important knowledge gap related to understanding the relative effectiveness of various heat mitigation agents.

Given the rising frequency of heat waves and shifting climatic patterns, there is a pressing need to develop heat-resilient strawberry production systems. This study was aimed to address these gaps by evaluating the effects of exogenous chemicals—abscisic acid, calcium chloride, kaolin, melatonin, and molasses on physiological, biochemical, and yield related traits in three strawberry genotypes under prolonged heat stress and to identify which genotype performs better. Therefore, it was hypothesized that exogenous application of physiological regulators would alleviate heat induced stress in strawberries by enhancing physiological resilience and yield performance in a genotype-dependent manner.

## Materials and methods

### Experimental site and duration

The study was carried out at the Department of Crop Botany and Tea Production Technology, Sylhet Agricultural University, Bangladesh (Latitude: 24.9 °N; Longitude: 91.8 °E; Elevation: ~9 m above sea level) from February 2024 to April 2025. This region, characterized by a humid subtropical climate with distinct seasonal variations, served as the experimental site for both field and laboratory phases.

### Experimental timeline

**Table:** 

Activity	Time period	Details
Pot culture initiation	February 2024	Pot experiment started using the same cohort of plants maintained throughout the study period
First fruiting and data collection	February–March 2024	Fruit data for the first cycle collected
Commencement of exogenous chemical treatments	Early March 2024	Treatments with ABA, CaC_2_, kaolin, melatonin, and molasses initiated
Continuation of treatments	March–May 2024	Spraying continued regularly during rising temperature period
Vegetative data collection	End of March, April, and May 2024	Plant growth and physiological traits recorded at three intervals
Treatment maintenance	June–November 2024	Treatments continued intermittently as needed throughout the year
Second fruiting and data collection	February–March 2025	Fruit data for the second growing cycle collected

### Climatic conditions

A digital data logger was installed to log real-time meteorological data, including temperature, humidity, and solar radiation. The experimental region typically experiences summer temperatures exceeding 30 °C, which posed natural heat stress conditions for the crop.

### Soil characteristics and pot preparation

Soil analysis revealed an acidic pH of 5.85 with 16.6% moisture content. Electrical conductivity was 21.4 µS cm^−1^ and total dissolved solids measured 14.5 mg L^−1^. Organic matter content was 2.8%. For uniform growth conditions, 54 pots (8 L capacity) were prepared with sieved hilly soil, amended with recommended fertilizers based on BARC^24^ 250 g vermicompost, 1.67 g triple superphosphate (TSP), 1.83 g muriate of potash (MoP), and 0.08 g magnesium sulfate (MgSO_4_). The lower 2 inches of each pot were layered with pebbles and sand to ensure adequate drainage and aeration.

### Experimental design

The experiment was designed as a two-factor Completely Randomized Design (CRD) with three replications per treatment, totaling 54 experimental units.

The two factors were:

Factor A: Strawberry Varieties.

V1: RABI-3.

V2: BARI Strawberry-2.

V3: BARI Strawberry-3.

Factor B: Exogenous Chemical Treatments.

T0: Control (Distilled water).

T1: Abscisic Acid (ABA) – 5 ppm.

T2: Calcium Chloride (CaCl_2_) – 10 mM.

T3: Kaolin – 5%.

T4: Melatonin-10 ppm.

T5: Molasses-5%.

The doses of exogenous chemicals were selected according to Salwa et al.^19^

### Plant material and propagation

Three locally developed strawberry cultivars were selected and collected (two from Bangladesh Agricultural Research Institute-BARI and one from a local nursery of Rajshahi) for their adaptability to the regional climatic conditions. Five-month-old runner plants were sourced in February 2024 from the departmental germplasm. These were propagated via vegetative runners in polybags under shaded conditions. Vigorous and healthy seedlings were transplanted into pots in the last week of February 2024. No approval is essential for conducting this research and even for collection of study plant materials.

### Fertilizer application

Supplemental fertilizer was applied 15 days after transplantation. Equal amounts of vermicompost and chemical fertilizers were administered per pot. A bio-organic supplement comprising mustard oil cake (fermented in water) was applied weekly around the plant base. At the reproductive stage, a nutrient solution was prepared by mixing 10 g each of urea, MoP, TSP, MgSO_4_, and vermicompost, along with 100 g mustard oil cake in 2 L water. The homogenized solution was applied to each pot for optimal fruiting.

### Agronomic practices

Weeding and Irrigation: Manual weeding was conducted as needed, concurrently loosening the soil to enhance root aeration. Irrigation was regulated based on soil moisture content.

Pest and Disease Management: Strawberry plants encountered biotic stress from mealybugs, aphids, caterpillars, and diseases such as fruit rot, leather rot, leaf scorch, and blight. Cypermethrin (synthetic pyrethroid) and elemental sulfur were applied at 30-day intervals for pest and disease control.

Protection Measures: Protective netting and polybags were employed during fruit ripening to prevent damage from birds, rodents, and insects.

### Exogenous chemical treatments

Treatments began in early March 2024, coinciding with rising day and night temperatures. ABA (T1), CaCl_2_ (T2), and Melatonin (T4) were prepared and stored under refrigeration. Similarly, kaolin (T3) and molasses (T5) were prepared at 5% concentrations using locally sourced materials. All solutions were brought to room temperature before application. Treatments were sprayed every three days until the plants were thoroughly soaked. The control group received distilled water in the same manner. Treatment application tapered after May due to seasonal rainfall but resumed intermittently until November, when ambient temperatures began to drop.

### Fruit harvesting

Fruit harvesting began in the first week of February for both 2024 and 2025. Ripening was characterized by progressive reddening from the base to the apex and the appearance of a glossy wax layer. Fruits with > 80% red surface coverage were considered mature for harvest.

### Data collection

*Vegetative stage* Morphological and physiological data were collected at the end of March, April, and May 2024 to assess plant responses across a gradient of heat stress.

*Reproductive stage* Yield and quality data were gathered post-harvest, from February to March 2025.

### Morphological parameters

Plant Height (cm): Measured from soil level to the terminal shoot using a tape measure.

Number of Leaves plant^−1^: Counted manually from each plant.

### Physiological parameters

Leaf Relative Water Content (RWC %): Determined using the method of Stocker (1929)^25^.

Leaf Pigments: Chlorophyll and carotenoid concentrations were measured spectrophotometrically following Porra et al.^26^.

### Biochemical parameters

Antioxidant Activity (% DPPH inhibition): Estimated using the free radical scavenging method with DPPH as per Kavaz and Faraj^27^.

Malondialdehyde (MDA, nmol g^−1^ FW): MDA levels were quantified to assess lipid peroxidation using a modified method from Debnath et al.^28^.

### Fruit quality assessment

Fruit Dimensions: Length and breadth (cm) were measured using a scale on three randomly selected fruits per treatment.

Fresh and Dry Weight (g fruit^−1^): Fresh fruits were weighed using a digital balance. Dry weight was recorded after oven-drying at 70 °C for 24 h.

Dry Matter Content (%): Calculated as:

Dry Matter (%) = (Dry Weight / Fresh Weight) × 100.

Vitamin B1 and B2 Content (mg 100 mL^−1^ FW): Estimated according to Fernandes et al.^29^.

Vitamin C Content (mg 100 mL^−1^ FW): Determined using the method of Salkic et al.^30^.

Antioxidant Activity of Fruits (%): Measured using the DPPH method, as per Kavaz and Faraj^27^.

### Yield attributes

Number of Fruits Plant^−1^: Counted from all plants during the harvest window.

Fruit Variability (g harvest^−1^): Fruits were harvested every alternate day from February 15 to March 13, 2025, and weighed.

Total Yield (g plant^−1^): Summation of all fruit weights plant^− 1^ over the harvest period. Total yield data were recorded on a plant^− 1^ basis due to the pot culture setup.

### Statistical analysis

Data were curated and processed using Microsoft Excel before analysis in R^31^ and RStudio (Version: 2025.09.2–418, URL-https://support.posit.co/hc/en-us/articles/206212048-Citing-RStudio)^32^. Means and standard deviations were computed from three replications per treatment. Two-way analysis of variance (ANOVA) was used to evaluate the significance of differences among treatments and varieties. Where significant differences were observed, mean comparisons were conducted using Duncan’s Multiple Range Test (DMRT) at the 5% significance level, following Gomez and Gomez ^33^.

## Results

### Average monthly air temperature (°C)

**Fig. 1:**
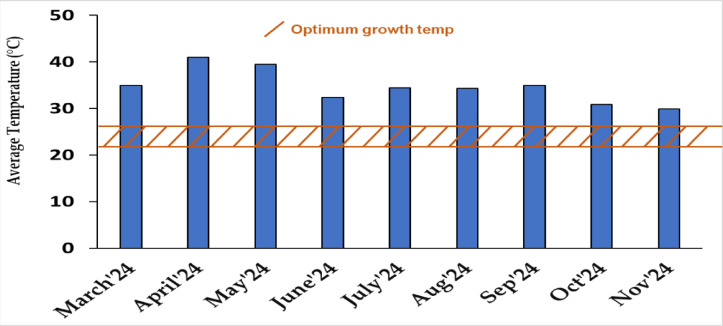
Average monthly air temperature between March, 2024 to November, 2024 at Sylhet.

During the study, average monthly air temperatures ranged from 29.95 °C to 41 °C (Fig. 1). The highest temperature was recorded in April 2024 (41 °C), followed by May (39.5 °C) and March (35 °C). Temperatures gradually decreased from June (32.42 °C) through August (34.26 °C), with the lowest averages observed in October (30.87 °C) and November (29.95 °C).

Agricultural University, Sylhet-3100, Bangladesh.

### Morphological growth

#### Plant height (cm)

**Fig. 2:**
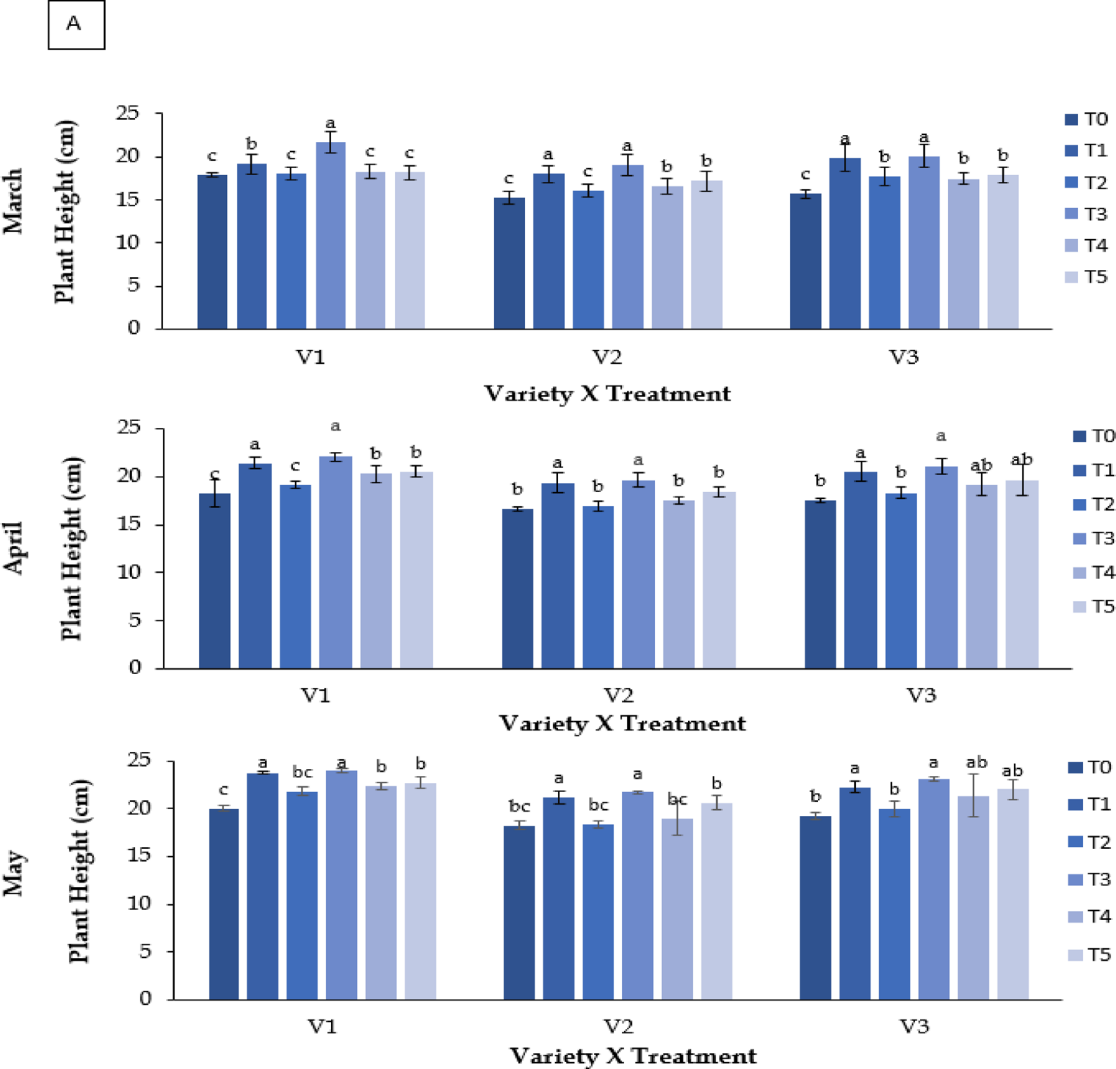

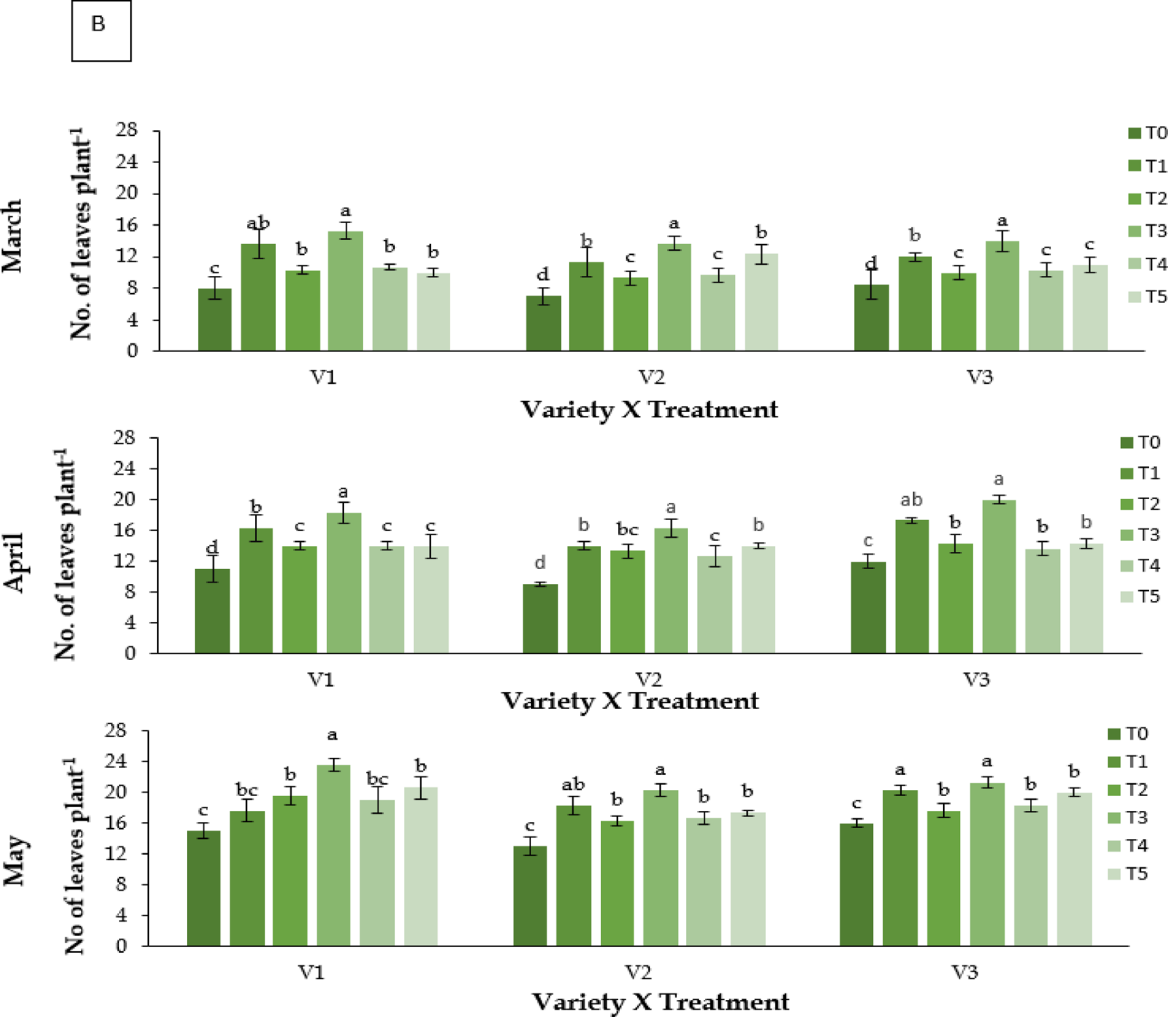
Effect of chemicals on (**A**) plant height (cm) of different Strawberry varieties and (**B**) number of leaves plan^1^ during March to May. Where, V_1_ = RABI-3, V_2_ = BARI Strawberry-2 and V_3_ = BARI Strawberry-3; T_0_ = Control, T_1_ = Abscisic acid, T_2_ = CaCl_2_, T_3_ = Kaolin, T_4_ = Melatonin and T_5_ = Molasses. In each variety, bars having common letter(s) do not differ significantly at *P* ≤ 0.05 as per DMRT.

A significant interaction was observed between strawberry genotype and treatment on plant height (*P* ≤ 0.05; Fig. 2A). Plant height increased from March to May across all genotypes, with variation in magnitude among treatments. RABI-3 (V1) had the greatest height under Kaolin (T3) in May (20.03 cm). BARI Strawberry-2 (V2) had the lowest height, particularly in the control (T0; 15.27 cm in March). Abscisic acid (T1) and Molasses (T5) produced moderate increases relative to control.

### Number of leaves plant^−1^

Significant differences were found among treatments and varieties (*P* ≤ 0.05; Fig. 2B). Leaf number increased progressively from March to May. Kaolin (T3) resulted in the highest leaf numbers, particularly in RABI-3 (V1; 15.33 to 23.67 leaves), while control plants had the fewest leaves.

#### Physiological growth of leaves

##### Leaf relative water content (% LRWC)

**Fig. 3:**
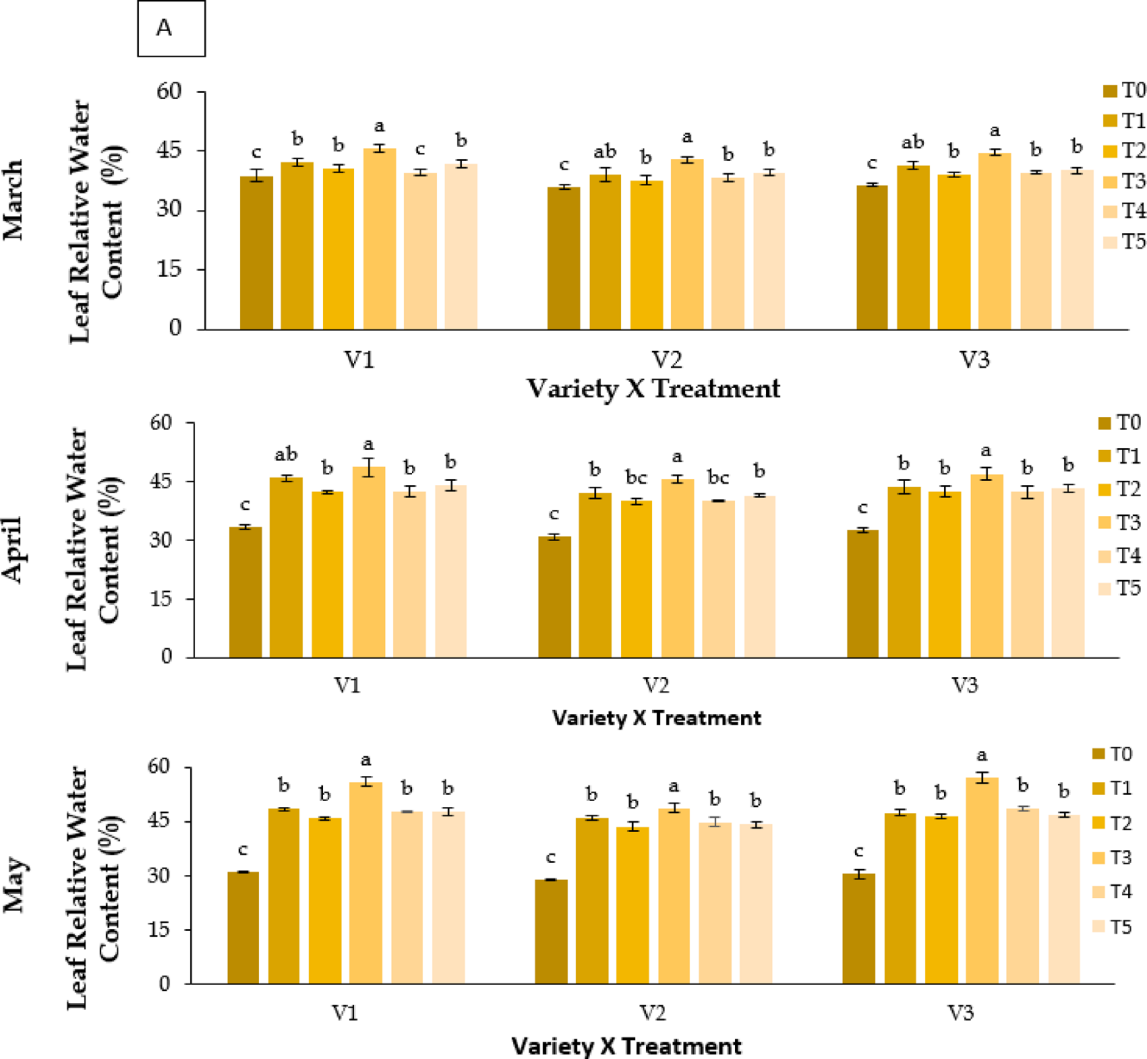

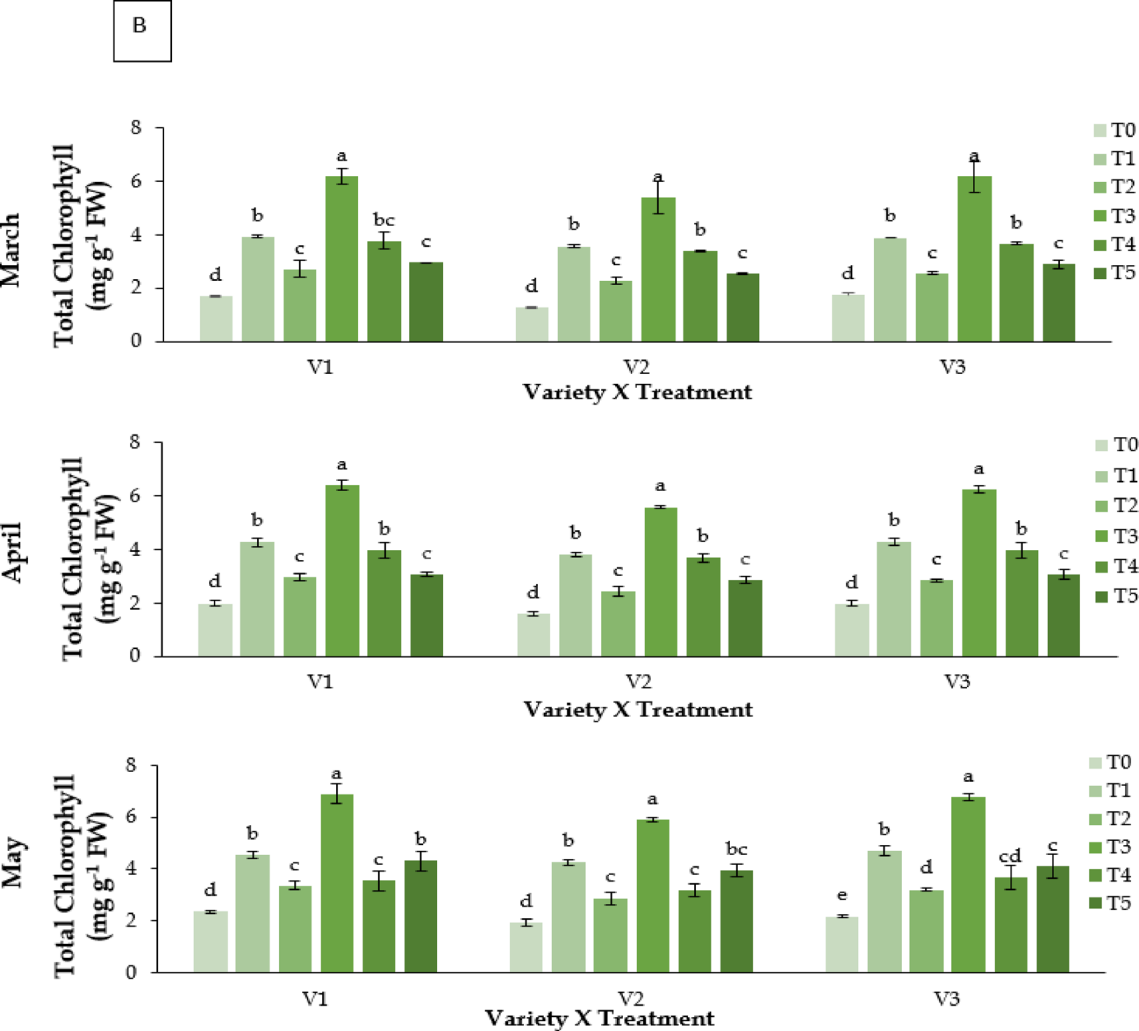

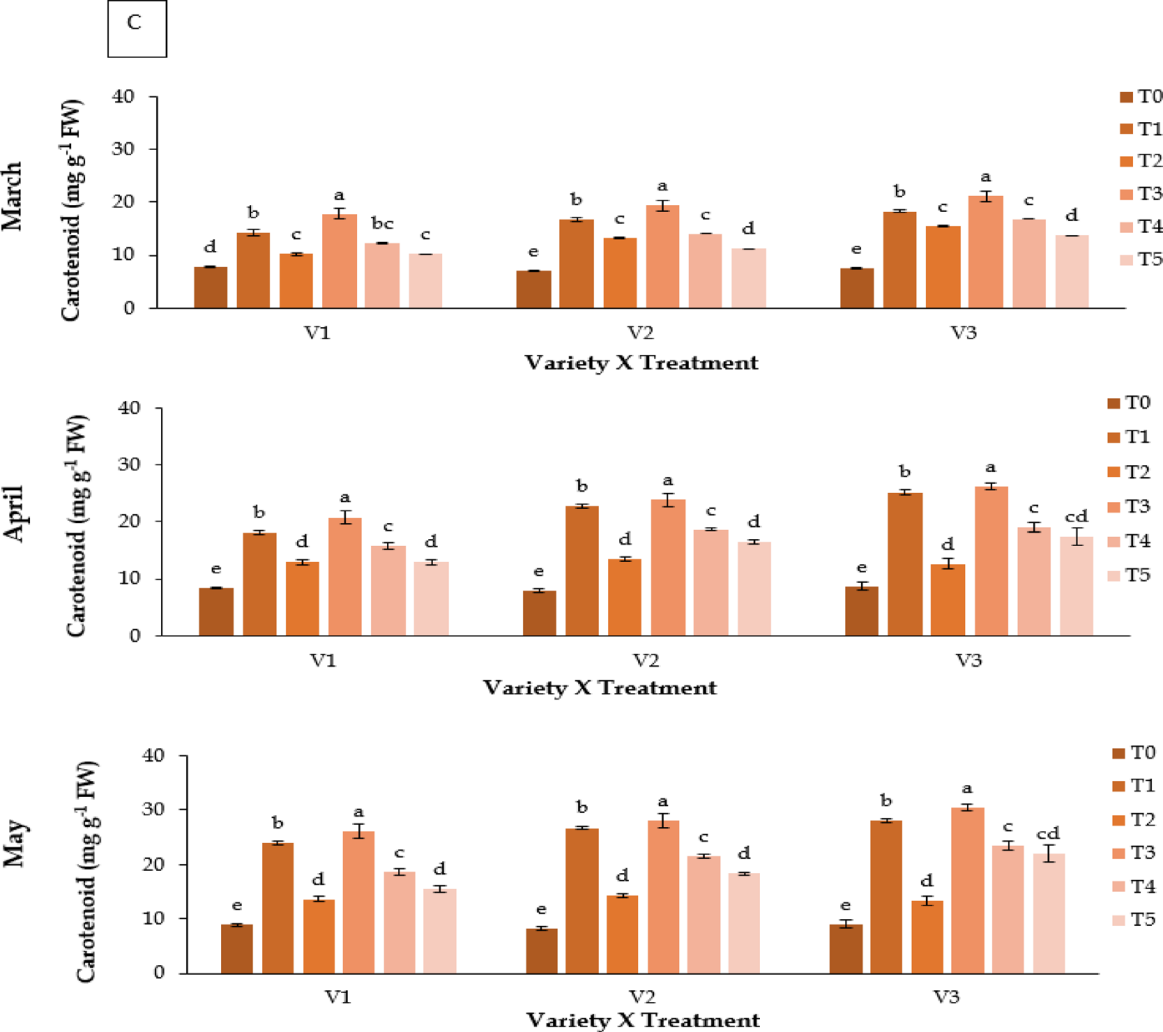
Effect of chemicals on (**A**) leaf relative water content (%), (**B**) total Chlorophyll content (mg g^− 1^ FW) and (**C**) carotenoid content (mg g^− 1^ FW) of different Strawberry varieties during March to May. Where, V_1_ = RABI-3, V_2_ = BARI Strawberry-2 and V_3_ = BARI Strawberry-3; T_0_ = Control, T_1_ = Abscisic acid, T_2_ = CaCl_2_, T_3_ = Kaolin, T_4_ = Melatonin and T_5_ = Molasses. In each variety, bars having common letter(s) do not differ significantly at *P* ≤ 0.05 as per DMRT.

Leaf relative water content varied significantly with treatment and genotype (*P* ≤ 0.05; Fig. 3A). Kaolin (T3) maintained the highest LRWC across varieties, peaking at 56.83% in BARI Strawberry-3 (V3) during May. RABI-3 (V1) showed 55.82% under T3, whereas BARI Strawberry-2 (V2) recorded the lowest values (48.55%). Control plants (T0) showed pronounced declines (28.89% in V2, May). The general ranking was T3 > T1 ≈ T5 > T2 ≈ T4 > T0.

#### Total chlorophyll content (mg g^− 1^ FW)

Chlorophyll content differed significantly among treatments and genotypes (*P* ≤ 0.05; Fig. 3B). Kaolin (T3) produced the highest chlorophyll content across varieties, peaking in May (V1: 6.912 mg g^−1^ FW; V3: 6.797 mg g^−1^ FW). Abscisic acid (T1) ranked second, while Control (T0) and CaCl_2_ (T2) recorded the lowest values. RABI-3 (V1) and BARI Strawberry-3 (V3) consistently showed higher chlorophyll concentrations than BARI Strawberry-2 (V2).

### Carotenoid content (mg g^− 1^ FW)

Carotenoid content varied significantly (*P* ≤ 0.05; Fig. 3C). Kaolin (T3) produced the highest carotenoid levels, particularly in V3 (30.48 mg g^−1^ FW in May). Control plants had the lowest carotenoid concentrations (7.12–9.05mgg^−1^FW).

### Biochemical study of leaves

#### Antioxidant activity (%)

**Fig. 4:**
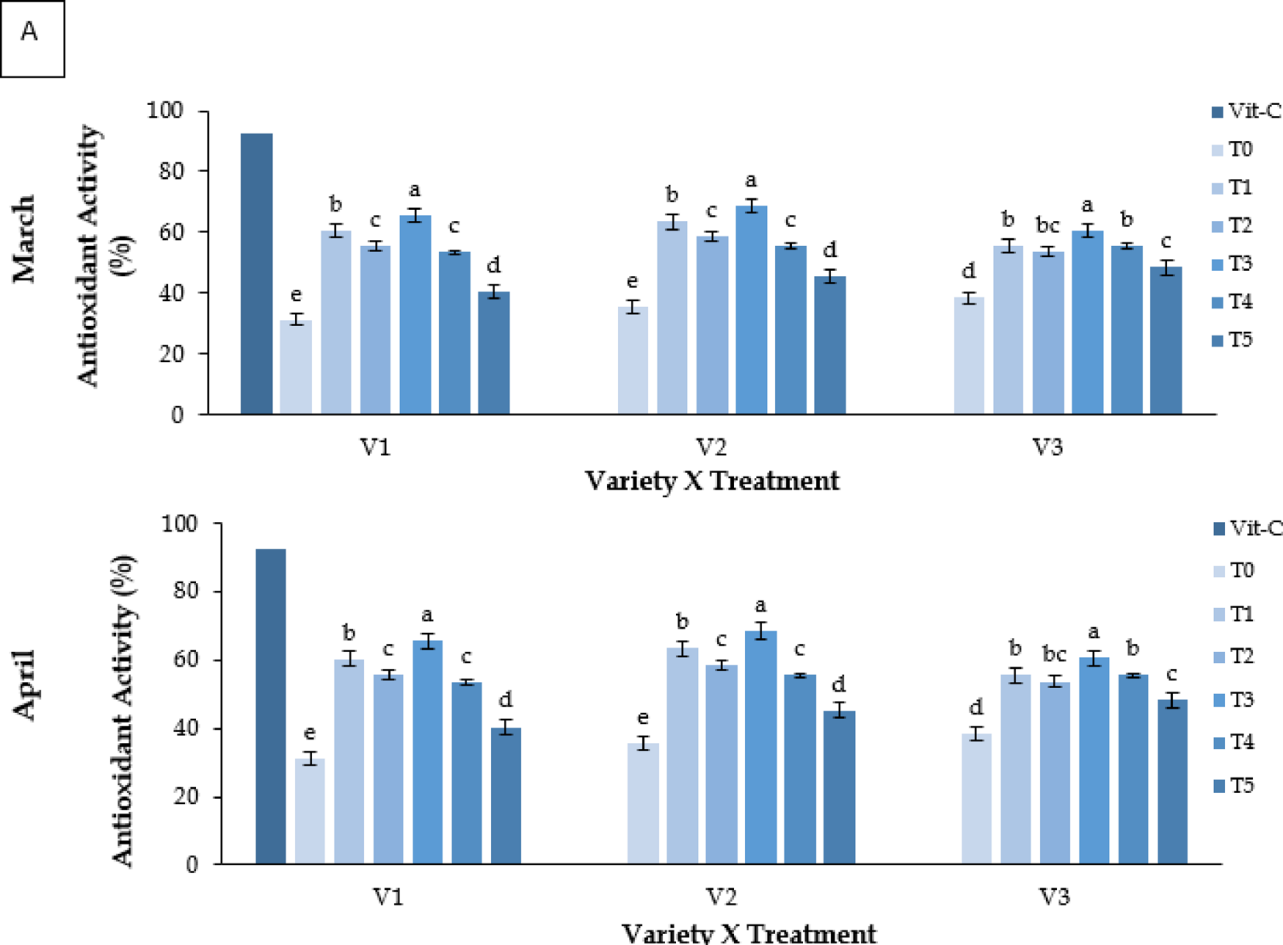

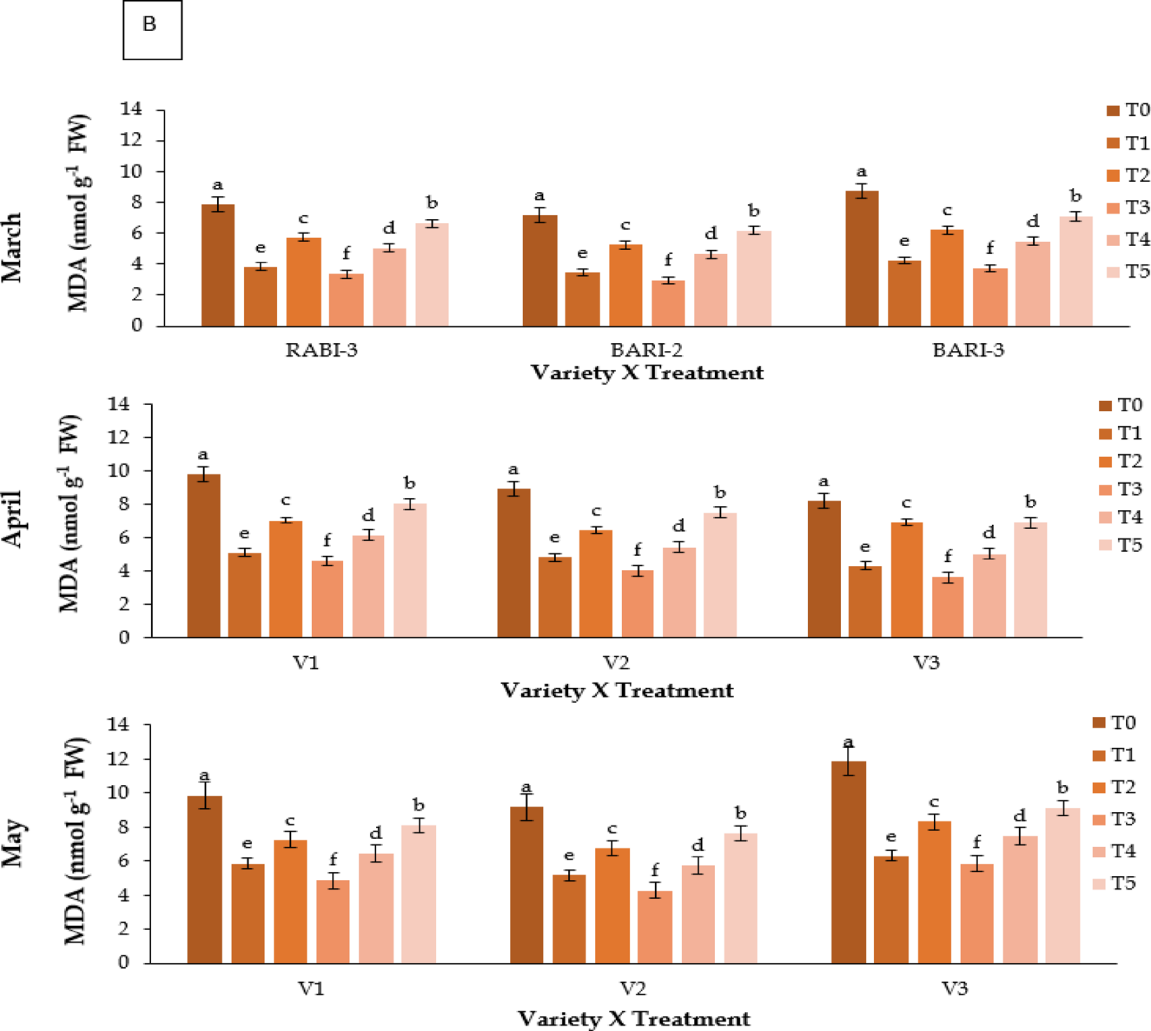
Effect of chemicals on (**A**) antioxidant activity (%) of different Strawberry varieties and (**B**) malondialdehyde (MDA) content (nmol g^− 1^ FW) during March to May. Where, V_1_ = RABI-3, V_2_ = BARI Strawberry-2 and V_3_ = BARI Strawberry-3; T_0_ = Control, T_1_ = Abscisic acid, T_2_ = CaCl_2_, T_3_ = Kaolin, T_4_ = Melatonin and T_5_ = Molasses. In each variety, bars having common letter(s) do not differ significantly at *P* ≤ 0.05 as per DMRT.

Antioxidant activity, measured by DPPH radical scavenging, differed significantly across genotypes and treatments (*P* ≤ 0.05; Fig. 4A). Kaolin (T3) yielded the highest antioxidant activity in all varieties. BARI Strawberry-3 (V3) consistently showed higher antioxidant levels. The standard vitamin C reference exhibited 90–93% activity.

### Malondialdehyde (MDA) content (nmol g^− 1^ FW)

MDA content, an indicator of lipid peroxidation, showed significant differences among treatments (*P* ≤ 0.05; Fig. 4B). Kaolin (T3) reduced MDA levels across all varieties, where V3 showed the lowest value (3.75 nmol g⁻^1^ FW). Control plants showed the highest values (up to 11.87 nmol g⁻^1^ FW).

### Fruit quality

#### Fruit dimensions

**Fig. 5:**
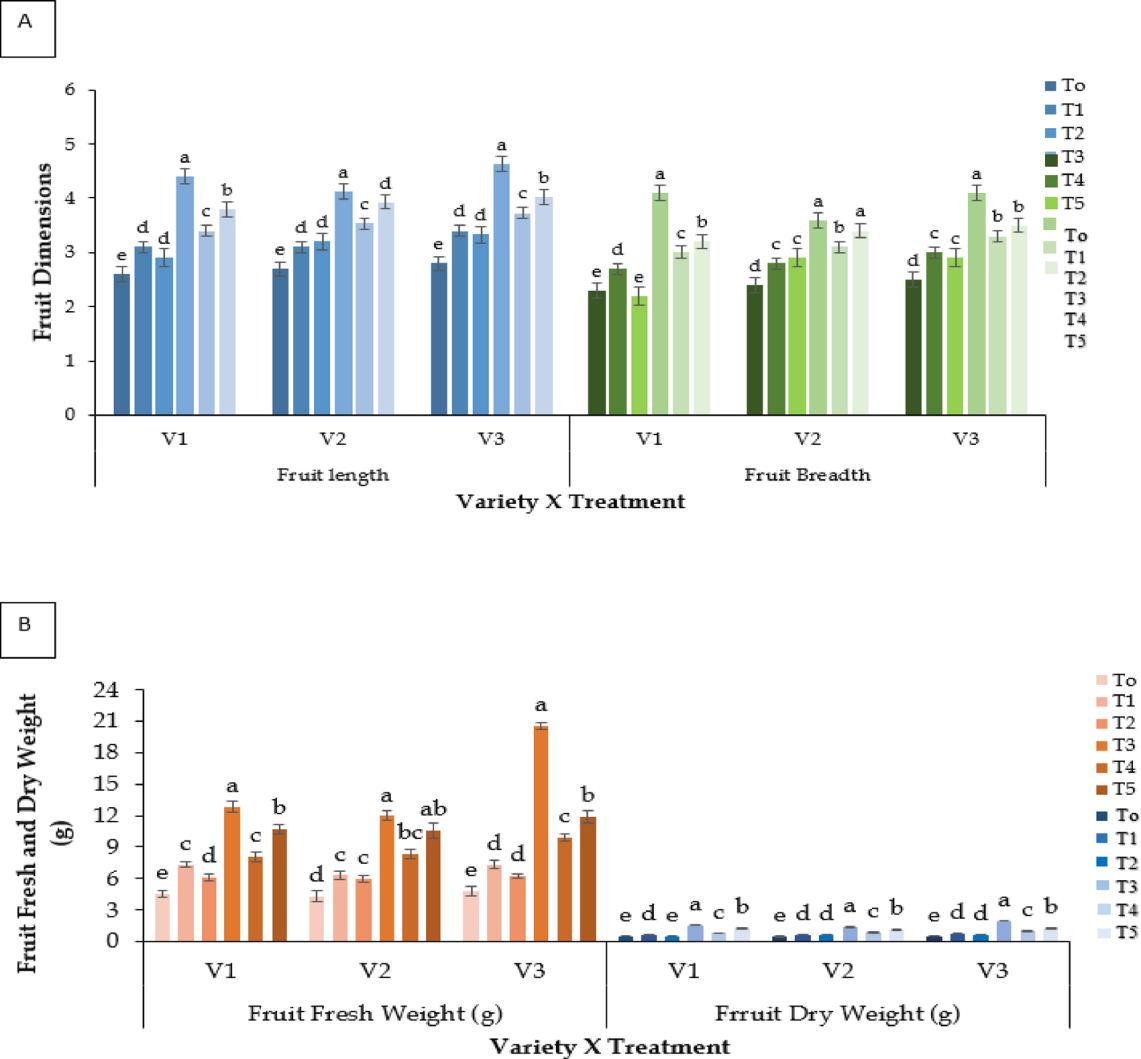

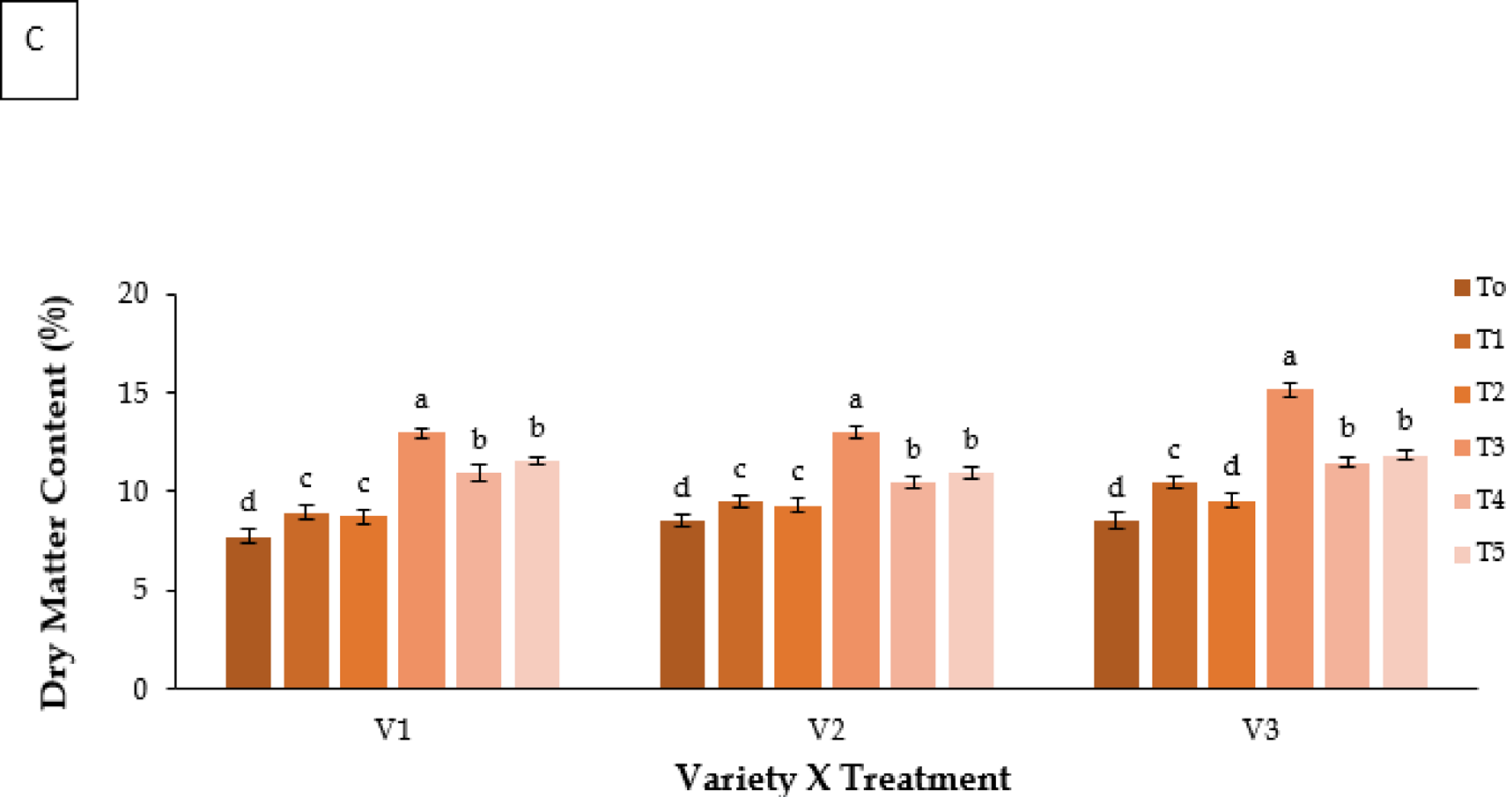
Effect of chemicals on (**A**) fruit length (cm) and fruit breadth (cm), (**B**) fruit fresh weight (g fruit^− 1^) and dry weight (g fruit^− 1^) and (**C**) dry matter content (%) of different Strawberry varieties. Where, V_1_ = RABI-3, V_2_ = BARI Strawberry-2 and V_3_ = BARI Strawberry-3; T_0_ = Control, T_1_ = Abscisic acid, T_2_ = CaCl_2_, T_3_ = Kaolin, T_4_ = Melatonin and T_5_ = Molasses. In each variety, bars having common letter(s) do not differ significantly at *P* ≤ 0.05 as per DMRT.

Fruit length and breadth were significantly affected by treatment and genotype (*P* ≤ 0.05; Fig. 5A). Kaolin (T3) improved both parameters, V3 reached 4.63 cm length and 4.13 cm breadth (approximately 65% higher than control). Molasses (T5) produced moderate effects.

#### Fruit fresh weight and dry weight (g fruit^− 1^)

Fruit weight varied significantly among treatments (*P* ≤ 0.05; Fig. 5B). Kaolin (T3) produced the largest fruits (V3: 20.51 g fruit^−1^), while control plants had the lowest (4.32–5.53 g fruit^−1^). Dry weight followed a similar pattern, with Kaolin (T3) showed the highest value (1.95 g fruit^−1^ in V3).

#### Fruit dry matter content (%)

Significant variation in dry matter content was observed among treatments (*P* ≤ 0.05; Fig. 5C). Kaolin (T3) resulted in the highest dry matter (15.17% in V3), followed by Molasses (T5). The general varietal order was V3 > V2 > V1.

#### Biochemical study of fruits

#### Total soluble solids (TSS)

**Fig. 6:**
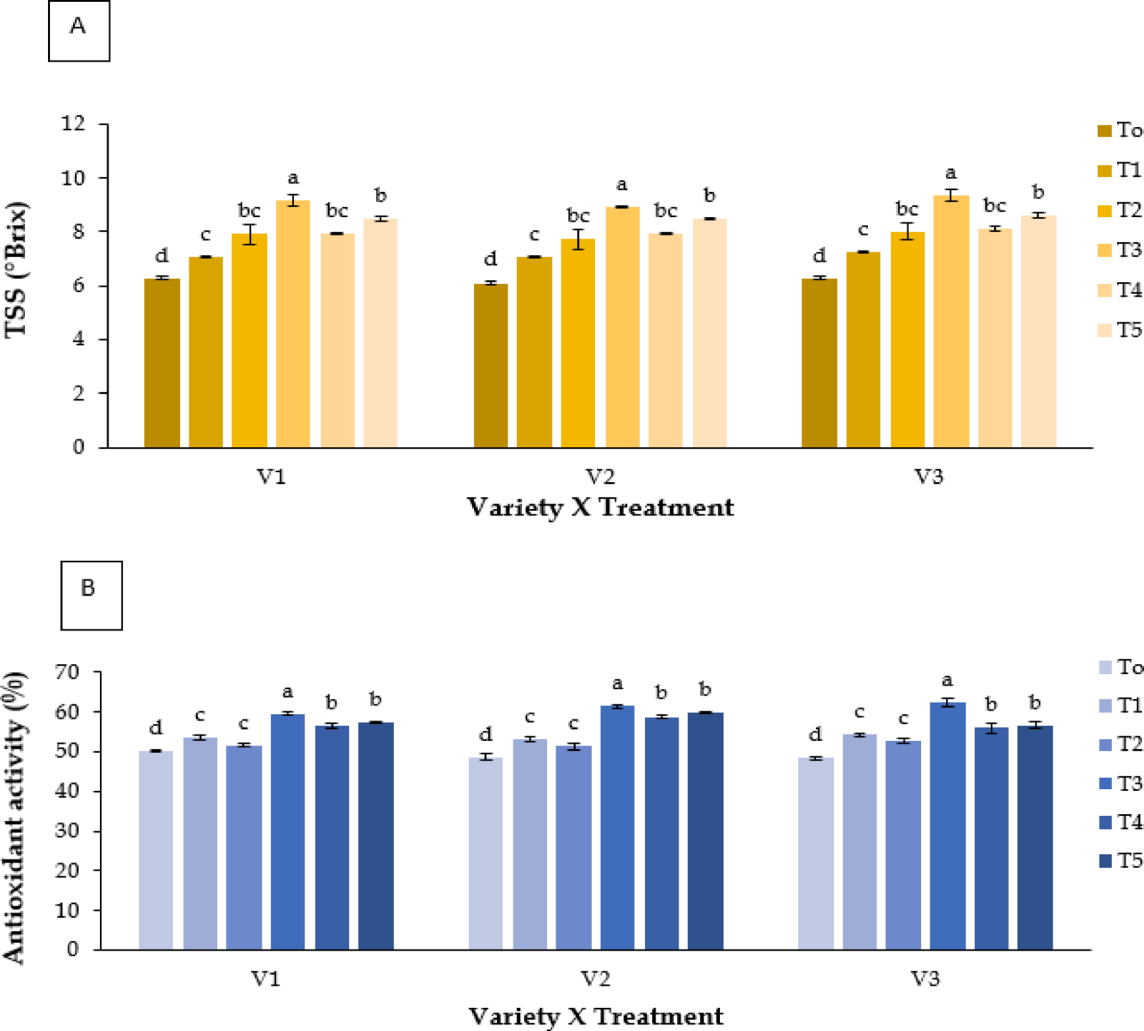

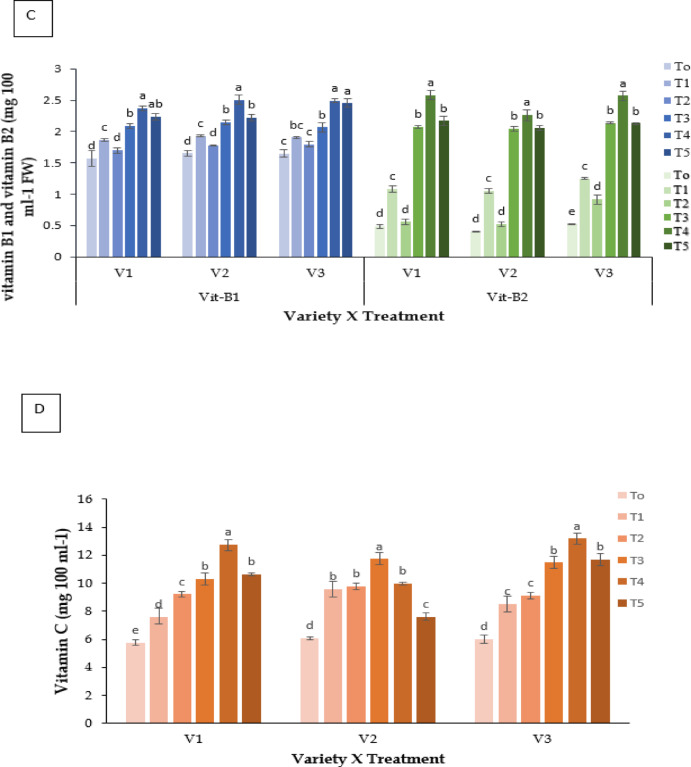
Effect of chemicals on (**A**) Total Soluble Solids (ºBrix), (**B**) antioxidant activity (%), (**C**) vitamin B1 and vitamin B2 (mg 100 ml^− 1^ FW), and (**D**) vitamin C (mg 100 ml^− 1^ FW) of different Strawberry varieties. Where, V_1_ = RABI-3, V_2_ = BARI Strawberry-2 and V_3_ = BARI Strawberry-3; T_0_ = Control, T_1_ = Abscisic acid, T_2_ = CaCl_2_, T_3_ = Kaolin, T_4_ = Melatonin and T_5_ = Molasses. In each variety, bars having common letter(s) do not differ significantly at *P* ≤ 0.05 as per DMRT.

TSS content differed significantly among treatments and varieties (*P* ≤ 0.05; Fig. 6A). Kaolin (T3) led to the highest TSS in all genotypes, reached 9.367 °Brix in V3.

#### Antioxidant activity (%) of fruit

Fruit antioxidant activity varied significantly (*P* ≤ 0.05; Fig. 6B). Kaolin (T3) resulted in the highest activity (V3: 62.36%), followed by Molasses (T5; 55.89–58.75%). Control plants showed the lowest activity (48–50%).

#### Vitamin B1 and B2 (mg 100 ml^− 1^ FW)

Vitamin B1 and B2 contents were significantly affected by treatment and genotype (*P* ≤ 0.05; Fig. 6C). Melatonin (T4) produced the highest values, with V2 recording 2.507 mg 100 ml^−1^ FW for vitamin B₁. Kaolin (T3) also improved vitamin concentrations compared with control.

#### Vitamin C (mg 100 ml^− 1^ FW)

Vitamin C levels differed significantly (*P* ≤ 0.05; Fig. 6D). Melatonin (T4) produced the highest concentrations (V3: 13.189 mg 100 ml^−1^ FW), followed by Kaolin (T3) and Molasses (T5).

### Yield traits

#### Number of fruits plants^− 1^

**Fig. 7:**
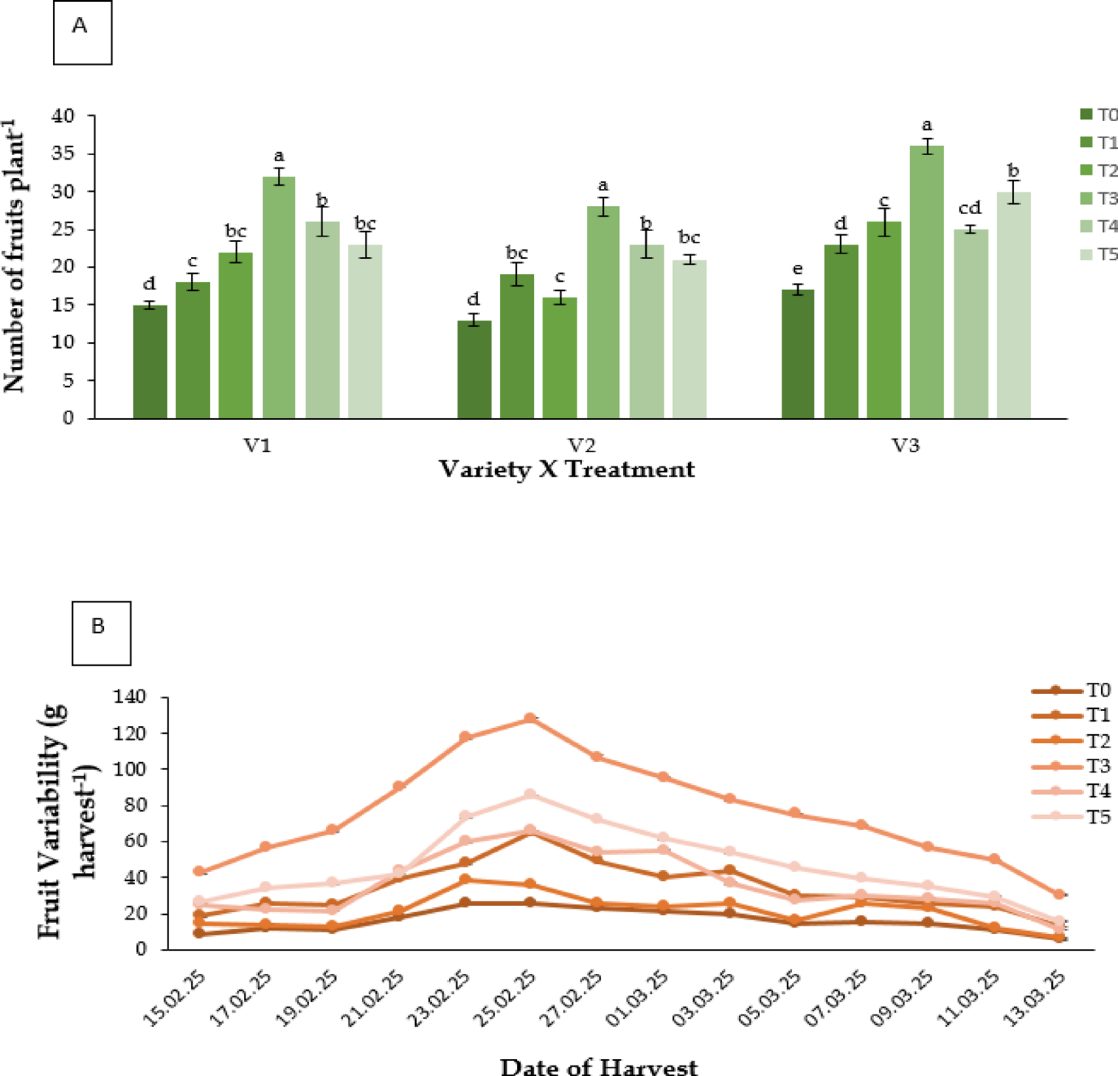

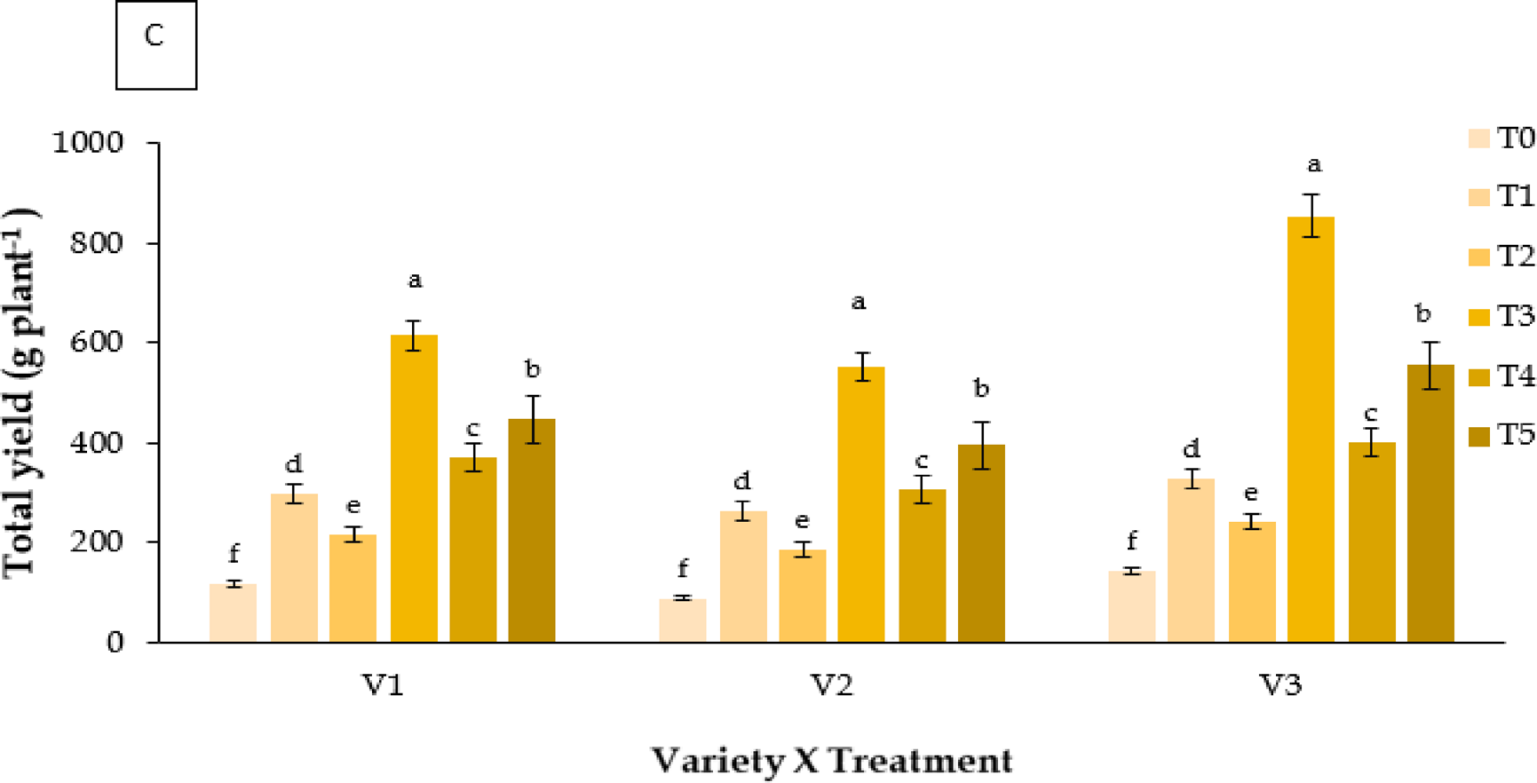
Effect of chemicals on (**A**) number of fruits plant^− 1^ of different Strawberry varieties, (**B**) fruit variability (g harvest^− 1^) production through one day interval under different treatments of three Strawberry varieties and (**C**) total yield (g plant^− 1^) of different Strawberry varieties. Where, V_1_ = RABI-3, V_2_ = BARI Strawberry-2 and V_3_ = BARI Strawberry-3; T_0_ = Control, T_1_ = Abscisic acid, T_2_ = CaCl_2_, T_3_ = Kaolin, T_4_ = Melatonin and T_5_ = Molasses. In each variety, bars having common letter(s) do not differ significantly at *P* ≤ 0.05 as per DMRT.

Fruit count per plant varied significantly among treatments (Fig. 7A). Kaolin (T3) produced the maximum number (V3: 36 fruits per plant), while control plants produced the fewest (17 fruits).

#### Fruit variability (g harvest^− 1^)

Kaolin (T3) also led to the highest yield per harvest across all dates (Fig. 7B)., peaking at 127.4 g harvest^− 1^. Yield improvements were most pronounced in late February, with all treatments outperforming control.

#### Total yield (g plant^− 1^)

Total fruit yield per plant differed significantly (*P* ≤ 0.05; Fig. 7C). Kaolin (T3) resulted in the highest yield (V3: 854.6 g plant^−1^), followed by RABI-3 (V1: 615.3 g plant^−1^). Control plants yielded the least.

#### Comparison of fruit quality traits seasons

Comparison across seasons showed that Kaolin (T3) improved fruit size, weight, and total soluble solids in 2025 compared with both control fruits in 2025 and baseline fruits from 2024 (Table 1). Vitamin C content under T3 was slightly lower than in 2024, but the difference was not statistically significant (*P* ≤ 0.05).

**Table 1:** Comparison of fruit quality traits of BARI Strawberry-3 between 2024 and 2025 Seasons.

Variety	Treatments	Fruit Length (cm)	Fruit Breadth (cm)	Fruit weight (g)	TSS (°Brix)	Vit-C (mg 100 ml⁻^1^ FW)
BARI Strawberry-3	Untreated fruits of 2024	3.68^b^	3.06^b^	14.76^b^	8.9^a^	12.05^a^
	Kaolin treated fruits of 2025	4.63^a^	4.12^a^	20.51^a^	9.36^a^	11.47^a^
	Controlled fruits of 2025	2.8^c^	2.5^c^	4.78^c^	6.24^c^	6.01^c^
Pr (> F)		0.0004	< 0.0001	0.00006	0.013	0.024

## Discussion

The present study evaluated the comparative efficacy of several exogenous treatments—abscisic acid (ABA), melatonin, molasses, and kaolin in mitigating heat-induced stress in different strawberry genotypes under controlled conditions. The findings revealed significant treatment-dependent differences in morphological, physiological, and yield-related traits, indicating varied thermotolerance mechanisms among genotypes. The results are discussed below in relation to previous studies, with emphasis on the physiological and agronomic implications of kaolin-mediated heat stress alleviation and its potential role in sustainable strawberry production under warming climates.

### Morphological responses

Plant height is among the earliest morphological indicators of thermal stress, as elevated temperature impairs cell division and elongation through hormonal imbalance, enhanced respiration, and oxidative injury^14^. In this study, the marked reduction in plant height across control plants (T0) confirmed the detrimental effect of heat on vegetative growth. Among the genotypes, BARI Strawberry-2 (V2) exhibited the lowest maximum height, indicating higher thermosensitivity. Kaolin application (T3) improved height and canopy structure, consistent with previous studies^34,35^ reporting its reflective and photoprotective film that reduces canopy temperature and prevents solar injury. The positive response under molasses and ABA treatments also reflects their reported roles in providing supplemental carbon sources^36^ and modulating stress-responsive growth pathways^37,38^.

Leaf production followed a similar pattern, declining sharply with rising temperature due to accelerated ethylene-mediated senescence. Kaolin markedly improved leaf number and canopy density, particularly in RABI-3 (V3), suggesting enhanced microclimate regulation and photoprotection^39,40^. These findings support earlier reports that kaolin improves light interception, reduces photoinhibition, and enhances canopy photosynthetic potential under stress.

### Physiological changes

Relative Water Content (RWC) declined in all genotypes under heat stress, indicating reduced cellular hydration and osmotic imbalance^40,41^. Control plants (T0) showed the greatest decline, particularly BARI Strawberry-2 (V2). Kaolin (T3) maintained the highest RWC, reflecting reduced transpirational loss due to canopy cooling. ABA (T1) and molasses (T5) also improved water status through enhanced osmotic adjustment and stomatal regulation^42,43^. ABA induced proline and soluble sugar accumulation likely contributed to improved turgor maintenance.

Heat stress also led to significant degradation of chlorophyll and carotenoids, consistent with earlier studies^14,44^. Kaolin preserved higher pigment levels, suggesting reduced photooxidative damage and sustained photosynthetic efficiency^21^. Oxidative stress, as reflected by elevated MDA levels, was most pronounced in control plants, whereas kaolin-treated plants showed reduced lipid peroxidation and higher antioxidant activity^15,23,45^. Melatonin (T4) and ABA (T1) also conferred moderate protection, though their effects may have been limited by compound instability under high light and temperature^46^⁻^50^.

Overall, kaolin provided superior physiological protection through multiple mechanisms reduction of canopy heat load, improved hydration, and stabilization of photosynthetic pigments supported by the activation of antioxidative defense pathways.

### Fruit quality and yield

High temperature during reproductive stages adversely affected fruit size, weight, and biochemical quality, particularly in BARI Strawberry-2 (V2). Kaolin treatment significantly improved fruit dimensions, weight, and total soluble solids (TSS), aligning with its positive impact on canopy cooling and assimilate partitioning^50^. ABA and molasses treatments moderately enhanced fruit size and sugar content through hormonal and osmotic regulation^51–53^.

Antioxidant activity and vitamin concentrations (B1, B2, and C) were highest under kaolin and melatonin treatments, indicating enhanced synthesis of bioactive compounds under mitigated oxidative stress ^[^⁴⁵, ⁴⁶^]^. The superior performance of BARI Strawberry-3 (V3) under kaolin treatment highlights its resilience and suitability for cultivation under thermal stress conditions.

### Practical and ecological relevance

Kaolin stands out for its eco-friendly and cost-effective nature. With a market price of approximately USD 12.27 per kg in Bangladesh, it is affordable and non-toxic to soil biota, pollinators, and groundwater systems^38^. Its inert, reflective coating offers sustainable climate adaptation potential for strawberry cultivation in heat prone regions.

## Conclusions

Kaolin spray (5%) proved to be the most effective treatment for mitigating heat stress in strawberry plants. It significantly enhanced vegetative growth, physiological stability, fruit quality, and yield. BARI Strawberry-3 demonstrated superior adaptability and productivity, confirming its potential for high temperature cultivation. The findings suggest that 5% kaolin foliar spray during peak summer can reduce thermal injury, preserve photosynthetic efficiency, and sustain yield.

The integration of kaolin-based management into tropical strawberry production systems offers an eco-safe and economically viable approach to climate resilience. Future research should combine kaolin with other organic or hormonal treatments to explore potential synergistic effects and further improve crop performance.

### Limitations and future directions

This study was conducted under a single growing season and environmental condition, which may limit the extrapolation of results to other climates or soil types. The limited replication size and environmental fluctuations could have influenced treatment responses. Moreover, only a selected set of foliar treatments and genotypes were evaluated, which restricts broader generalization. Future studies should include multi-season, multi-location trials and molecular analyses to confirm treatment effects under diverse stress conditions.

Future investigations should focus on:

- Field scale validation across multiple agro-ecological zones and growing seasons.
- Molecular and physiological characterization to identify genes and signaling pathways involved in heat tolerance.
- Optimization of melatonin and ABA dosages under field conditions to overcome compound instability.
- Economic feasibility assessments and farmer participatory trials to evaluate cost effectiveness and scalability for sustainable commercial production.

Sadia Shabnam Swarna = Data curation, Data analysis, Manuscript writing and editing.

Prof. Dr. Sharifunnessa Moonmoon = Conceptualization, Methodology Investigation. Supervision, Validation, Funding, Project administration, Resources, Manuscript writing and editing.

Funding.

Ministry of The Science and Technology, Government of the Peoples Republic of Bangladesh [grant numbers: SRG-142197; 2024-25], and Sylhet Agricultural University Research System supported this study. But there is no fund or support is available regarding this article submission, processing and publishing from that or any other national or international funding organization.

All data generated or analyzed during this study are included in this manuscript. Other’s will be provided on request from the corresponding author.

## Data Availability

All data generated or analyzed during this study are included in this manuscript. Other’s will be provided on request from the corresponding author.

## Abbreviations

ANOVA: Analysis of variance
BARC: Bangladesh Agricultural Research Council
BARI: Bangladesh Agricultural Research Institute
CaCl_2_: Calcium chloride
Cm: Centimeter
CRD: Completely randomized design
et al.: and his associated
Fig.: Figure
FW: Fresh weight
G: Gram
LSD: Least significant differences
MDA: Malondialdehyde
MgSO_4_: Magnesium sulfate
MOP: Muriate of potash
Mg: Milligram
Nmol: Nanomoles
Ppm: Parts per million
ROS: Reactive gen spices
LRWC: Leaf relative water content
TSP: Triple superphosphate
TSS: Total soluble solids
°C: Degree celsius
Plant^− 1^: Per plant
d^− 1^: Per day
mL^− 1^: Per milliliter
